# Closing the loop on glioblastoma: A roadmap toward developing bioelectronics for continuous monitoring of tumor state

**DOI:** 10.1063/5.0312207

**Published:** 2026-03-12

**Authors:** Ester Clarisse do Couto Lopes, Joshua P. A. Daoud, Alexandra Collisson, Ariadni Georgiannakis, Joshua Killilea, Cédric M. John, Dimitrios Paraskevopoulos, Christopher A. R. Chapman

**Affiliations:** 1 School of Engineering and Materials Science, Queen Mary University of London, London, United Kingdom; 2 Blizard Institute, Queen Mary University of London, London, United Kingdom; 3 Department of Bioengineering, Imperial College London, London, United Kingdom; 4 Digital Environment Research Institute, Queen Mary University of London, London, United Kingdom; 5 Department of Neurosurgery, The Royal London Hospital, Barts Health NHS Trust, London, United Kingdom

## Abstract

Closed-loop bioelectronic devices offer a promising platform for responsive treatment to heterogeneous disease states. Glioblastoma, an aggressive form of brain cancer, has recently emerged as a focus of bioelectronic medicine through delivery of electrotherapies. This perspective article posits that true progress in the management of this extremely heterogeneous disease requires the integration of continuous monitoring from the tumor microenvironment as well as on-device analytics to enact closed-loop control. Four promising candidate biological changes present in the glioblastoma microenvironment are highlighted (local field potentials, bioimpedance, local pH, biomarkers) alongside the bioelectronic sensors that can enable the development of multifunctional bioelectronic devices to monitor the changes. Finally, three key principles (patient involvement, data analytics, and device fabrication) governing the successful implementation of closed-loop sensors are proposed to create a roadmap for academics and industry partners to successfully develop multimodal devices for the treatment of glioblastoma.

## I. INTRODUCTION

Recently, bioelectronic devices have garnered significant attention for their potential use in the treatment of glioblastoma through application of electric fields.^1^ Tumor treating fields (TTFs) have been shown to improve survival outcomes in patients with glioblastoma, both in conjunction with temozolomide delivery^2^ and post-adjuvant therapy delivery.^3,4^ Additionally, irreversible electroporation (IRE) has shown some effectiveness in *in vitro* model settings.^5^ The development of new methods for treatment of brain cancers with active implantable devices has made cancer bioelectronics an exciting area in new medical device development. Much of this excitement stems from the promise of improved patient specific treatment and minimized off-target effects that personalized implanted devices offer.

Glioblastoma is an extremely invasive disease with much of its poor prognosis stemming from high intra-tumor heterogeneity.^6^ Heterogeneous disease states remain a significant barrier to the long-term success of active implantable bioelectronics as most active implantable devices are unable to react dynamically to changes in the surrounding disease state.^7^ The development of closed-loop devices holds promise for combining continuous monitoring of the surrounding tissue with direct modulation via a single active device.^8^ Unfortunately, in practice long-term monitoring of disease state has proven challenging.^9^

Thus far, the major focus in the development of bioelectronic devices for the modulation of glioblastoma has been on new therapeutic outputs (such as TTFs and IRE). This Perspective investigates the feasibility in closing the loop for these devices to offer true patient specific responsive treatment. Given the intrinsic intratumoural heterogeneity in glioblastoma, until there is a clear way forward to continuously monitoring the tumor state and adjusting treatment in real time, cancer bioelectronics will see extremely limited impact. Four promising measurable physiological signals that are implicated in glioblastoma infiltration may provide a way forward for developing devices that incorporate continuous monitoring with therapeutic output. Building on these signals, we propose a roadmap for practically incorporating multiple bioelectronic sensor types addressing issues such as sensor fabrication, data analytics, and patient input.

## II. MONITORING PHYSIOLOGICAL CHANGES IN GLIOBLASTOMA TUMOR ENVIRONMENT

In glioblastoma, there has been an impressive amount of previous research into potential methods for intraoperative tumor identification due to challenges involved in surgical tumor margin detection.^10^ Electrical activity, bioimpedance, local pH, and biomarker detection stand out as having potential for continuous monitoring of tumor state.

### A. Electrical activity

#### 1. The signal

**FIG. 1.**
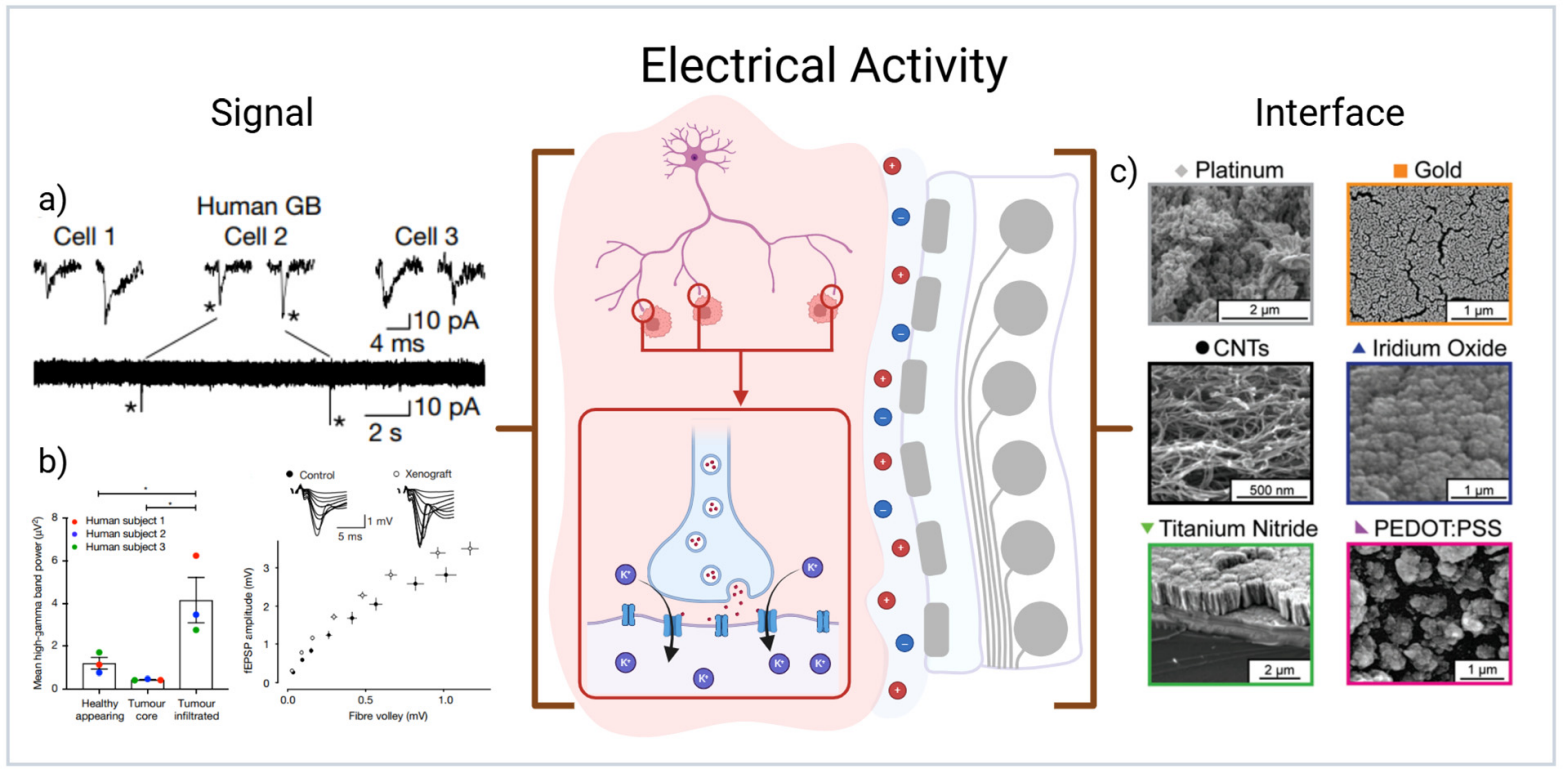
(a) Measurements of spontaneous excitatory postsynaptic currents (sEPSCs) recorded from three human glioblastoma cells demonstrating existence of neurogliomal synapses. Reproduced with permission from Venkataramani *et al.*, Nature **573**, 532–538 (2019). Copyright 2019 Springer.^14^ (b) Demonstration of high gamma band power shifts in LFP recordings of human glioblastoma patients with respect to healthy appearing tissue, and hyperexcitability captured in mouse xenograft models of glioblastoma. Reproduced with permission from Venkatesh *et al.*, Nature **573**, 539–545 (2019). Copyright 2019 Springer.^13^ (c) Scanning electron micrographs of common nanostructured material interfaces that promote low impedance and improved cellular coupling. Reproduced with permission from Chapman *et al.*, Adv. Funct. Mater. **28**, 1703523 (2018). Copyright 2018 Wiley.^9^ Created in BioRender, C. Chapman (2026) https://BioRender.com/pirdptb.

There is clear evidence that synaptic transmission onto cancer cells drives proliferation in both the central and peripheral nervous system.^11,12^ As high-grade glioblastoma integrates into neural circuits, the cancerous cells form synapses with neurons and respond to depolarizing signals that promote tumor growth via activity-dependent potassium currents and membrane depolarization^13,14^ [Fig. 1(a)]. It has been demonstrated that the interaction of cancer with healthy neurons shift the healthy neural populations into a measurable hyperexcitable state through imbalances in local ionic concentrations.^14^ This hyperexcitability leads to increased firing frequency in otherwise healthy neuron populations as the cancer cells actively seek the synapse of glutamate to drive proliferation through activation of AMPA receptors. This in turn drives lasting changes to the cell membrane potential and induces inward calcium flow and a significant increase in gamma band power^14^ [Fig. 1(b)].

Although these shifts have predominantly been measured through intracellular techniques such as patch clamp, the measurement of local field potential (LFP) activity from the tumor site presents a significant opportunity for high performance electrodes to continuously monitor for aberrant activity.^15^ Clinical outcomes of LFP recording in patients with glioblastoma are varied in their outputs however there is a consensus in the clinical literature that glioblastoma tissue exhibited a clear increase in frequencies relating to a stimulus.^16^ Of particular interest are some clinical studies reporting an increase in high gamma power^15,17^ such as reported in previous patch clamp studies^13,14^ by using LFP recording modalities.

#### 2. The interface

After surgical resection of glioblastoma tumors, the tumor margin is sparsely populated by residual tumor cells. Implanted neural electrodes have seen a step-change in resolution over the past decade, with the ability to resolve near single unit populations on an implanted device;^18^ however, maintaining high signal fidelity at high resolution needs close physical coupling at the electrode-cell interface.^19^

Increased physical coupling of cells has been investigated primarily through the addition of physical or chemical cues to the electrode surfaces. The most promising physical cue is the incorporation of nanostructured material surfaces onto the electrode sites. These physical cues have shown significant improvements for long-term physical coupling of neurons.^20^ Materials ranging from nanoporous metals,^21^ templated nanowires,^22^ and conjugated polymers^23^ have all shown significant promise [Fig. 1(c)]. Conversely, chemical surface cues such as binding motifs^24^ have also shown promise when used on easily functionalized substrates such as gold or crosslinked hydrogel systems. In addition to physical coupling, LFP recordings are highly dependent on positioning of the electrode arrays. As the resolution increases on implanted arrays, issues like micromotion induced signal drift become increasingly challenging to combat.^25^ One potential method for improving adhesion to the tumor resection cavity is through adding physical and/or chemical cues to the array insulation to further increase active coupling of the device to the tissue.

### B. Bioimpedance

#### 1. The signal

**FIG. 2.**
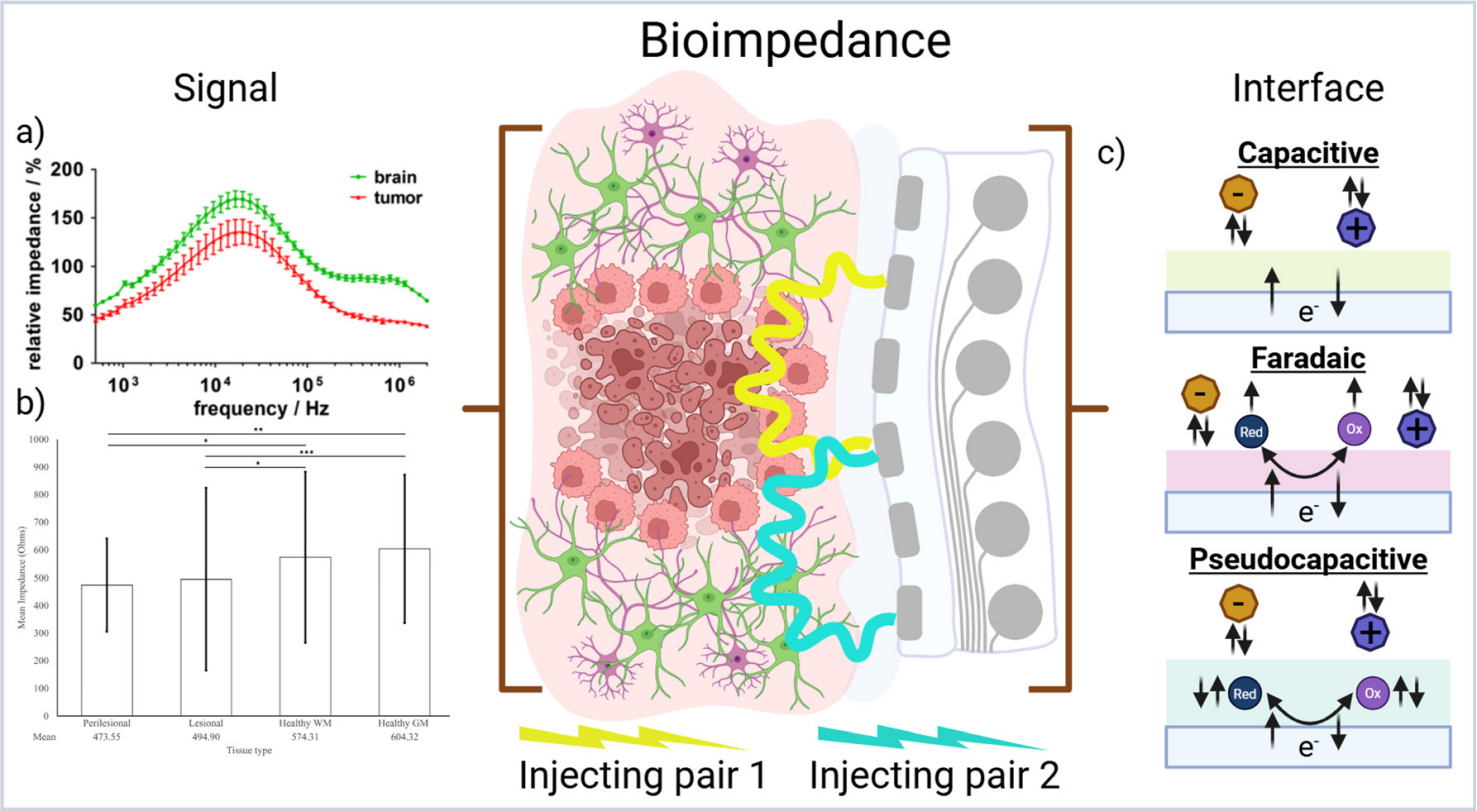
(a) Baseline corrected impedance spectra differences between healthy and cancerous brain tissue showing largest difference centered around 20 kHz. Reproduced with permission from Jahnke *et al.*, Biosens. Bioelectron. **46**, 8–14 (2013). Copyright 2013 Elsevier.^28^ (b) Mean values of clinically reported impedance measurements from perilesional, lesional, healthy white matter, and healthy gray matter (left to right). Reproduced with permission from Georgiannakis *et al.*, Neurosurg. Rev. **47**, 888 (2024). Copyright 2024 Springer.^16^ (c) Schematic depicting different charge transfer mechanisms on neural electrodes. Created in BioRender, C. Chapman (2026) https://BioRender.com/rwfolnq.

Tumor tissues often differ from healthy tissues in terms of ionic concentration and water content,^26^ which leads to altered electrical conductivity and, consequently, changes in impedance.^27^ Glioblastoma has been shown to have higher conductivity (i.e., lower impedance) than healthy tissue.^28^ These differences form the basis for using bioimpedance measurements as a diagnostic tool in oncology. Several studies have reported significant distinctions in the electrical properties of tumor vs normal tissues, supporting its potential for cancer detection^29–31^ [Fig. 2(a)]. The clinical use of bioimpedance measurements has also shown clear trends in the reported values for lesional and healthy tissues^16^ [Fig. 2(b)]. There are however significant challenges in the measurement of tissue impedance. As bioimpedance is the result of physical tissue composition, ionic concentration, and electrode contact impedance care must be taken when interpreting bioimpedance differences to ensure they are not caused by non-biological changes (i.e., motion and electrode-tissue interface).^32^

#### 2. The interface

Most of the clinical implementations of bioimpedance measurements have used probe electrodes with high impedance tips inserted directly into the tumor mass rather than flat electrocorticography (ECoG) arrays. When measuring impedance from ECoG arrays, the material selection is important. Electrode interface material used must be carefully selected to reduce time lag (i.e., phase shift) between the electrodes driving current flow and the measuring electrodes. In practice, this process favors materials that transfer charge through faradaic reactions as this purely resistive charge transfer minimizes phase shift^33^ [Fig. 2(c)]; however, many faradaic materials are unsuitable for biological measurement due their propensity to corrode during charge transfer.^34^ Recently, the benefits of the mixed mode conduction of conjugated polymer systems such as poly(3,4-ethylenedioxythiophene) (PEDOT), have highlighted significant improvements for measurement of bioimpedance.^35^

### C. Local pH

#### 1. The signal

Glioblastoma primarily relies on aerobic glycolysis for ATP production.^36^ The combination of low blood supply and glycolytic metabolism leads to the accumulation of lactic acid, creating an acidic tumor microenvironment.^36,37^ This acidification promotes neo-angiogenesis^38^ and enhances tumor invasiveness.^36^ Meanwhile, the alkaline intracellular pH of the cancer cells has been associated with reduced apoptotic activity.^39^ The extracellular microenvironment of glioblastoma cells *in vitro* is typically in the region of pH 6.1 to 6.8 with this level remaining constant throughout cell proliferation and cancer progression.^40^ *In vivo* intracellular changes in pH have been reported to increase from pH 7.01 in healthy cells to a mean of 7.05 in the whole tumor volume with a slightly elevated yet non-significant further increase to 7.07 in the necrotic core.^39^

The measurement of *in vivo* intracellular pH is accomplished through imaging techniques such as 31P magnetic resonance spectroscopy (MRS).^39–41^ As MRS necessitates the use of an MRI this method can be prohibitively expensive for more frequent checks and is unreasonable for use in a continuously monitoring paradigm. Other pH measurement techniques of the tumor microenvironment have primarily relied on low resolution electrochemical detection.

#### 2. The interface

Electrochemical measurements are particularly challenging to reproduce in the form factor of micro-scale systems as they necessitate the use of a reference electrode configuration. One of the most promising approaches for microscale pH measurement has been through the use of organic electrochemical transistors (OECTs) where changes in the gate dielectric modulate output current in response to ionic imbalances at the surface of a conjugated polymer layer between source and drain electrodes.^42^

**FIG. 3.**
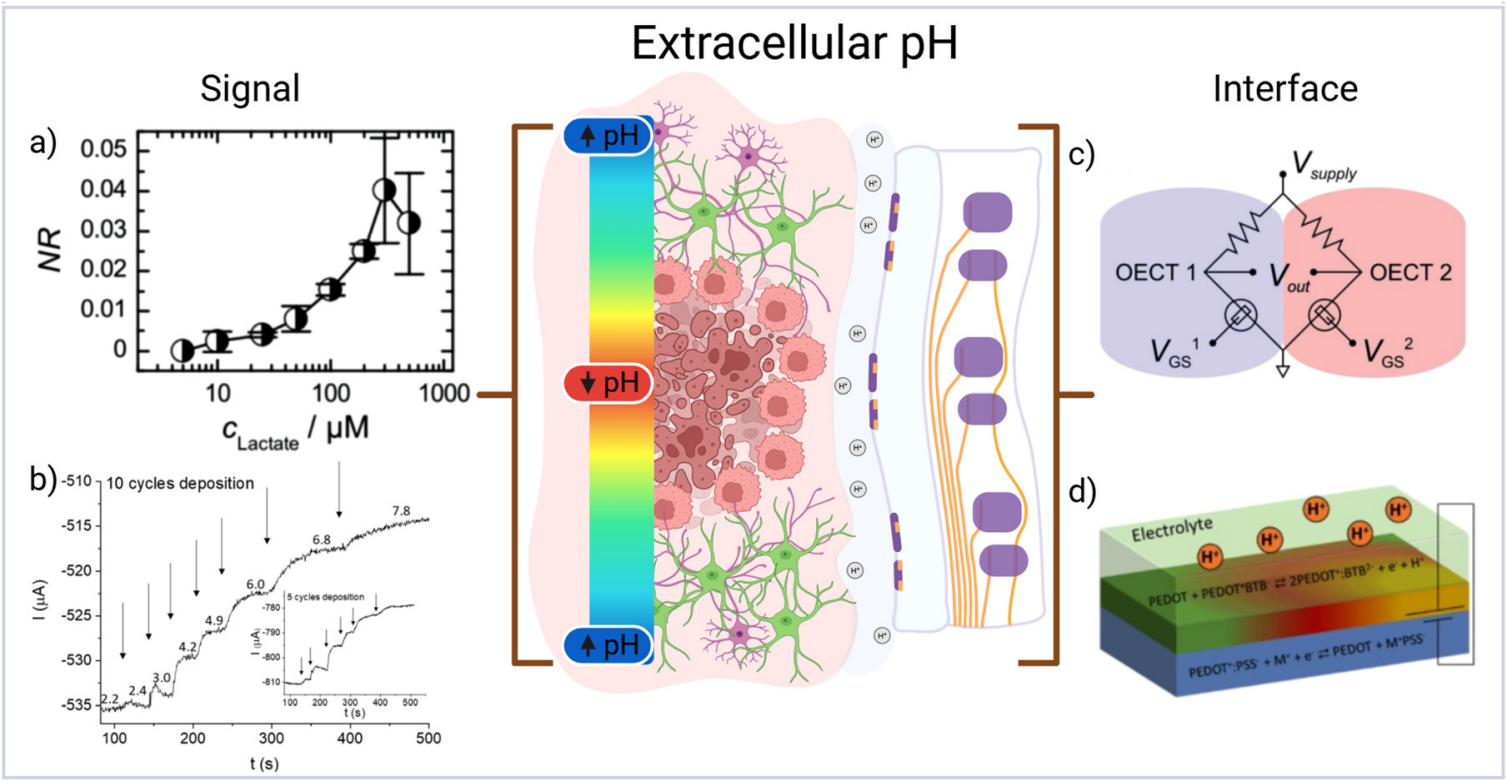
(a) Standard curve response of lactate oxidase functionalized OECT circuit showing large range of detection. Reproduced with permission from Braendlein *et al.*, Adv. Mater. **29**, 1605744 (2017). Copyright 2017 Wiley.^43^ (b) Response of pH increases from a PEDOT:BTB functionalized OECT circuit showing clear differentiation in glioblastoma relevant ranges. Reproduced with permission from Mariani *et al.*, Electrochem. Commun. **116**, 106763 (2020). Copyright 2021 Elsevier.^44^ (c) Electrical circuit diagram showing the Whetstone bridge configuration of OECTs for monitoring lactate concentration. Reproduced with permission from Braendlein *et al.*, Adv. Mater. **29**, 1605744 (2017). Copyright 2017 Wiley.^43^ (d) Schematic showing layering of PEDOT:PSS and PEDOT:BTB on a simple OECT circuit for pH sensing. Reproduced with permission from Mariani *et al.*, Electrochem. Commun. **116**, 106763 (2020). Copyright 2021 Elsevier.^44^ Created in BioRender, C. Chapman (2026) https://BioRender.com/8jn820b.

OECTs provide a flexible configuration that can be used to measure many different changes in the ionic environment that a semiconducting layer of polymer is exposed to. This can be done either through a multi OECT configuration (such as a Whetstone bridge circuit [Fig. 3(c)])^43^ or reformulating conjugated polymer chemistry to facilitate reference free measurements. By incorporating lactate oxidase enzyme into the polymeric semiconductor, the multi OECT configuration demonstrated efficient and sensitive measurements of lactate in cell culture media [Fig. 3(a)].^43^ Further improvements to pH sensitive OECTs have also been introduced by using ion sensitive polymers such as PEDOT doped with bromothymol blue (BTB) [Fig. 3(d)] enabling the measurement of pH without Ref. 44 [Fig. 3(b)]. Although this comes with a loss of sensitivity, changing chemistry can enable easier fabrication of the OECTs. Given the pronounced spatial heterogeneity of the tumor microenvironment, a single OECT is unlikely to reflect overall pH dynamics. Consequently, the use of distributed or array-based OECT networks is necessary to obtain a more comprehensive picture of intertumoral pH changes. This introduces specific challenges in the electronics needed to drive and measure signal from the OECTs. However, to this end, recent reports have demonstrated the potential for creating high density OECT arrays for brain recording.^45^

### D. Biomarker detection

#### 1. The signal

Biomarkers are molecules either produced by a tumor or resulting from the body's physiological response to the tumor. They play critical roles in early cancer screening, auxiliary diagnosis, prognosis prediction and monitoring response to treatment.^46^ In the context of implanted biosensors, not all biomarkers are suitable targets for biosensor development. An ideal biomarker must possess a suitable binding mechanism that can be effectively leveraged by the sensor. Furthermore, the biosensor must have adequate sensitivity in range of the biomarker's concentration in the intended biofluid. These limitations mean that the chronic measurement of tumor specific biomarkers using implanted electrodes is by far the most challenging physiological change to measure in glioblastoma.

Although certainly a challenge, recent reviews have highlighted many potential biomarkers that are overexpressed during glioblastoma progression.^47^ Two exciting target molecules that are abundantly overexpressed in glioblastoma tumors are interleukin 1 beta (IL-1β) and lipoprotein receptor protein 1 (LRP1). IL-1β is an inflammatory cytokine that is upregulated during glioblastoma progression.^48^ A recent study using implanted micro dialysis probes identified an approximate sixfold increase in IL-1β in the glioblastoma tumor microenvironment compared to surrounding healthy tissue.^49^ Similarly, LRP1 is a membrane protein involved in the transport of cholesterol and plays a significant role in glioblastoma aggressiveness.^50^ Recent studies comparing mRNA expression for LRP1 has demonstrated a significant approximately 10-fold increase from glioblastoma tissue compared to healthy tissue in patient samples.^50^ It is important to note that many glioblastoma biomarkers, including inflammatory cytokines such as IL-1β, are not tumor-specific and fluctuate in response to general neuroinflammation.^51^ As the post-resection tumor margin is in an extremely inflamed state this represents a challenge for the clinical translation of any single-analyte sensing approaches such as IL-1β since the sensors could saturate before the neuroinflammation abated.

#### 2. The interface

**FIG. 4.**
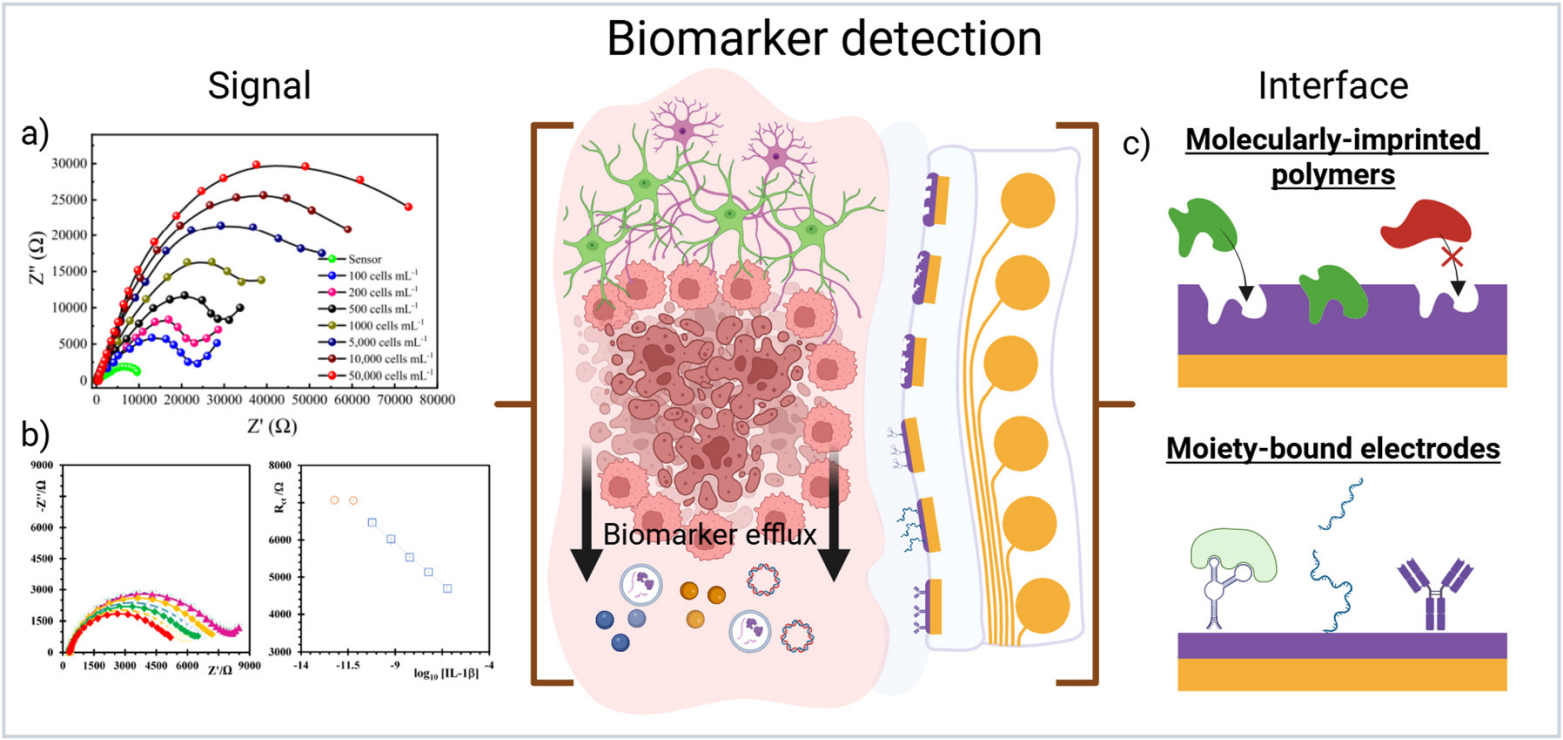
(a) Electrochemical impedimetric measurements of glioma cells by a moiety-bound electrode consisting of capturing protein angiopep-2. Reproduced with permission from Ganganboina *et al.*, Biosens. Bioelectron. **181**, 113151 (2021). Copyright 2021 Elsevier.^52^ (b) Electrochemical impedance shifts measured from an IL-1β molecularly-imprinted polymer electrode showing picomolar sensitivity. Reproduced with permission from Cardoso *et al.*, Bioelectrochemistry **130**, 107287 (2019). Copyright 2019 Elsevier.^57^ (c) Schematic showing differences between MIP and moiety-bound electrodes and their mode of detection. Created in BioRender, C. Chapman (2026) https://BioRender.com/q9woxaz.

There are many strategies for the detection of biomarkers using functionalized electrodes. Many of these strategies employ bespoke material systems and complex chemistry that would hinder production of clinically translatable bioelectronic devices.^52^ Two techniques stand out as easily translatable for incorporation into multimodal arrays are surface moiety-bound electrodes and molecularly-imprinted polymers (MIPs) [Fig. 4(c)].

Moiety-bound electrodes take advantage of bound electrochemical reporters that change state after capture of the desired biomarker.^53,54^ These biosensors typically rely on shape conformations or size exclusion to move redox reporting probes such as ferrocyanide or methylene blue toward or away from the electrode surface resulting in measurable electrochemical differences [Fig. 4(a)]. Gold electrodes, using thiol chemistry, are the exemplar for successful translation into usable biosensors, however identification or synthesis of capturing moieties limit application in implanted biosensors.

MIPs are made through the templating of a target biomarker into a polymer film or nanoparticle during polymerization. After polymerization, the biomarker is washed out leaving voids in the surface the polymer surface.^55^ These voids directly impact electrical continuity in the substrate, therefore, when exposed to target analyte the voids are filled leading to a measurable shift in electrical properties^56^ [Fig. 4(b)]. Additionally, their production is a relatively straightforward fabrication process and can, at least in theory, be used for any structure of analyte due to the imprinting process.

### E. Signal comparison

Each of the physiological signals discussed above offer valuable biological insight but their practical implementation varies considerably when taking into account their technological maturity and their distinct temporal scales. Table I provides a summary of the key characteristics and practical constraints associated with each signal proposed.

**TABLE I.** Comparison of sensing strategies for implantable glioblastoma monitoring devices. Modalities are evaluated according to their biological rationale, current technological maturity, practical device requirements, feasibility of implantation, expected signal stability, characteristic timescales, and major barriers to clinical translation.

Signal	Biological relevance	Practicality and maturity	Device requirements	Implantation feasibility	Signal stability	Temporal scale	Translational barriers
Electrical Activity	Reflects the interaction between healthy cells and cancer cells	High	Recording electrode, low-noise amplifier	Well established (ECoG)	Stable	milliseconds-seconds	Signal-to-noise ratio
Bioimpedance	Sensitive to tissue composition	High	Current injection and recording electrode	Feasible with existing technologies	Affected by encapsulation	seconds-minutes	Encapsulation and glial scar interference
Local pH	Reflects acidic tumor microenvironment	Moderate	Ion-sensitivity, OECT arrays	Technically feasible but limited clinical validation	Susceptible to drift	seconds-minutes	Spatial heterogeneity
Biomarkers	Tumor specific physiological response	Low	Surface moiety-bound electrodes or MIPs	Technically demanding	Unstable	minutes-hours	Biofouling, slow latency

## III. CONCEPTUAL CHALLENGES IN DEVELOPING IMPLANTED MULTIMODAL SENSOR ARRAYS

In heterogenous diseases such as glioblastoma monitoring, a single physiological change is unlikely to accurately predict the disease state. Additionally, differences in temporal scales between glioblastoma recurrence happening over days to weeks and signals changing in milliseconds to hours means that closing the loop with these signals requires more processing than simply measuring physiological signals. Moving forward, the development of these devices will require a focus on the consolidation and monitoring of multiple sensor types on a single implanted device. There are three major conceptual challenges to accomplishing this: sensor fabrication, data analytics, and patient impact.

### A. Sensor fabrication

Practical limitations in electrode implementation and fabrication can be overcome through design and selection of highly functional electrode materials. In this space, conjugated polymer systems have firmly cemented their place as one of the most multifaceted materials to use for multimodal neural electrodes. In particular, the conjugated polymer PEDOT has been used successfully in various forms. Taken together, these devices have shown conceptually that PEDOT as a fundamental building block to a neural interface has the potential be used to monitor multiple physiological changes associated with glioblastoma. PEDOT is not the only material capable of producing multimodal arrays, however the body of work that exists on conjugated polymer functionalized devices, and particularly PEDOT, is a natural starting point.

### B. Data analytics

A primary bottleneck of the implementation of multimodal sensor arrays is the collation and validation of the combined data into a data stream capable of informing a decision.

#### 1. Training models to use data from multisensor arrays

The data collected from the different probes will be of similar modality (i.e., a time series of electric voltage) and evaluated continuously. Machine learning using data streaming is a natural choice for this task, however, since the system would rely on continuous probe data, the model developed would need to be different than what is currently available.^58^ There are some specific challenges to overcome for in-patient machine learning systems.

First, the continuous time series of sensor output will need to be binned into discrete voltage values. If small differences in the time step exists between sensors, there is a risk of comparing signals recorded at different times. Once this challenge is resolved a machine learning model can be trained on the data. An offline solution, where the data are downloaded to a local machine for training, is easiest. Training the algorithm will require ensuring enough labeled data are collected. The truth value in this case could be e.g., presence or absence of high gamma activity (classification task), the concentration of key biomarkers or pH value (regression task), and ultimately presence (classification) and severity (regression) of glioblastoma. This represents a challenge because clinical studies do not typically comprise thousands of patients with and without glioblastoma, offering a rich collection of training data. Previous work has demonstrated that large datasets are needed for scientific machine learning;^59^ however, one way to overcome this is by developing *in vitro* models or using synthetic data.^60^

The necessary system will not be a single model but rather a series of machine learning models. For instance, one model could be tasked to determine the presence/absence of key biomarkers, whereas another model will integrate the output of model #1 with pH estimates to predict the severity of glioblastoma. The exact choice of models and their arrangements is a design choice dictated by the task and the nature and amount of data at hand. Some promising methods to facilitate decision making from the output of machine learning models are anomaly detection,^61^ trend-based prediction,^62^ or adaptive thresholding.^63^ In anomaly detection, a model output can be set to react to spikes (i.e., anomalies) in the model inputs. In practice, anomaly detection has a fast response time but the potential to react to signal changes not necessarily correlated with the disease state. Deploying adaptive thresholding can aid in the identification of aberrant activity caused by disease state as the magnitude of anomaly treated as an actionable signal can be altered in real time to address potential signal shifts from electrode encapsulation, movement, etc. The power of using a series of machine learning models with multimodal input means that anomaly detection of fast changing signals such as LFPs or biomarker concentration can be integrated into slower changing signal outputs such as bioimpedance and pH. Opposed to anomaly detection, trend-based prediction uses past data to inform a decision or “forecast” a state. While this type of analysis requires longer time to produce a predicted state, it has significant potential to match the timescale of glioblastoma recurrence. The decision pathway for detecting glioblastoma recurrence will likely succeed by deploying faster anomaly-based detection paradigms for individual signal inputs and deliver therapy based off a slower trend-based assessment of the tumor margin state.

The third challenge in patient monitoring is in the deployment of the model which will need to be deployed on edge (i.e., on the array itself). This is particularly important for glioblastoma monitoring because of how aggressive the disease is, and to reduce the burden of frequent doctor visits on both the patient and medical system. On edge deployment is challenging since the model will need to handle low power, and the need to transmit only small data packages.^64^ Hence, smaller models (statistical machine learning model, or “distilled” deep learning models^65^) would be preferred.^66^

#### 2. Developing *in vitro* models for data collection

Making a multisensor array for monitoring tumor state requires testing on a suitable biological model to collect and validate the sensor outputs. This is particularly challenging in the case of glioblastoma as continuous monitoring from post-surgical resection site using *in vivo* models is costly and time consuming. Instead, moving toward complex *in vitro* models may provide a much more efficient way to collect the large datasets needed to validate the combination of data streams.

Newer *in vitro* cancer models have started to include microfluidic technologies, which allows for precise fluid control, enabling closer replication of the environmental conditions of a tumour.^67^ Microfluidic systems offer high levels of customization and versatility, with the ability to integrate biosensors, which could be used to further enhance validation of implantable biosensors.^67,68^ Utilization of an *in vitro* model which combines patient specific cells with microfluidics could facilitate real-time recording of many biosensor parameters in a physiologically accurate environment, to enable patient specific optimization of implantable biosensors before clinical testing.

While 3D *in vitro* models more closely mimic tumor microenvironment than traditional 2D cultures, they still fall short of fully replicating human *in vivo* tumors, particularly with respect to the vascularity and the body's dynamic immune response.^69,70^ Nevertheless, they have the potential to provide a valuable starting point for mechanistic predictions and the validation of in multisensor array performance under controlled, physiologically relevant conditions.

### C. Patient impact: Learning from other implanted devices

Many implanted devices have forgone patient involvement in the development of treatments which has led to reduced patient uptake and device failure on a conceptual level. Complications with implanted neural devices are common, including infection, implant hardware issues, plus cognitive and neuropsychiatric disturbances.^71,72^ The high likelihood of complications adds significant patient concern about the long-term effects associated with implantable devices, which is exacerbated by the lack of clear post-trial management for implants studied in clinical trials.^73^

These concerns have led researchers to highlight the importance of obtaining patient-centered outcome measures and embedding feedback into implantable device development so that devices can better reflect patient preferences concerning implant size, location and style of external hardware.^74^ Due to the reduced life expectancy and high disease burden of glioblastoma, less invasive devices have also been explored, for example the Optune system, which delivers tumor treating fields (TTF).^75,76^ Despite being less invasive and boasting high rates of treatment compliance, recent studies have indicated that device features such as weight and appearance are limitations in terms of patient satisfaction.^77,78^ In that sense, implantable devices have significant potential to provide progress without adding excessive extra surgical steps in the glioblastoma treatment pipeline.

Ultimately, to overcome these limitations it is crucial that patients receive appropriate consideration in device design and development, to pave the way for more accessible and effective technologies. There must be a shift toward patient centric device development if bioelectronic implants are going to see widespread use. There is progress with many agencies requiring well thought out patient and public interaction and engagement (PPIE) provisions in new projects, however, there needs to be a bigger mentality shift in the neurotechnology field to consider patient perception and experience before designing highly invasive implanted devices.

## IV. COMPLETING THE LOOP

The space surrounding neurotechnology development has been rapidly expanding with dedicated research programs being funded alongside a thriving startup community. The crosscutting of neurotechnology into the field of cancer treatment and diagnosis offers a paradigm shifting opportunity to improve the treatment and overall quality of life to patients with glioblastoma.

**FIG. 5.**
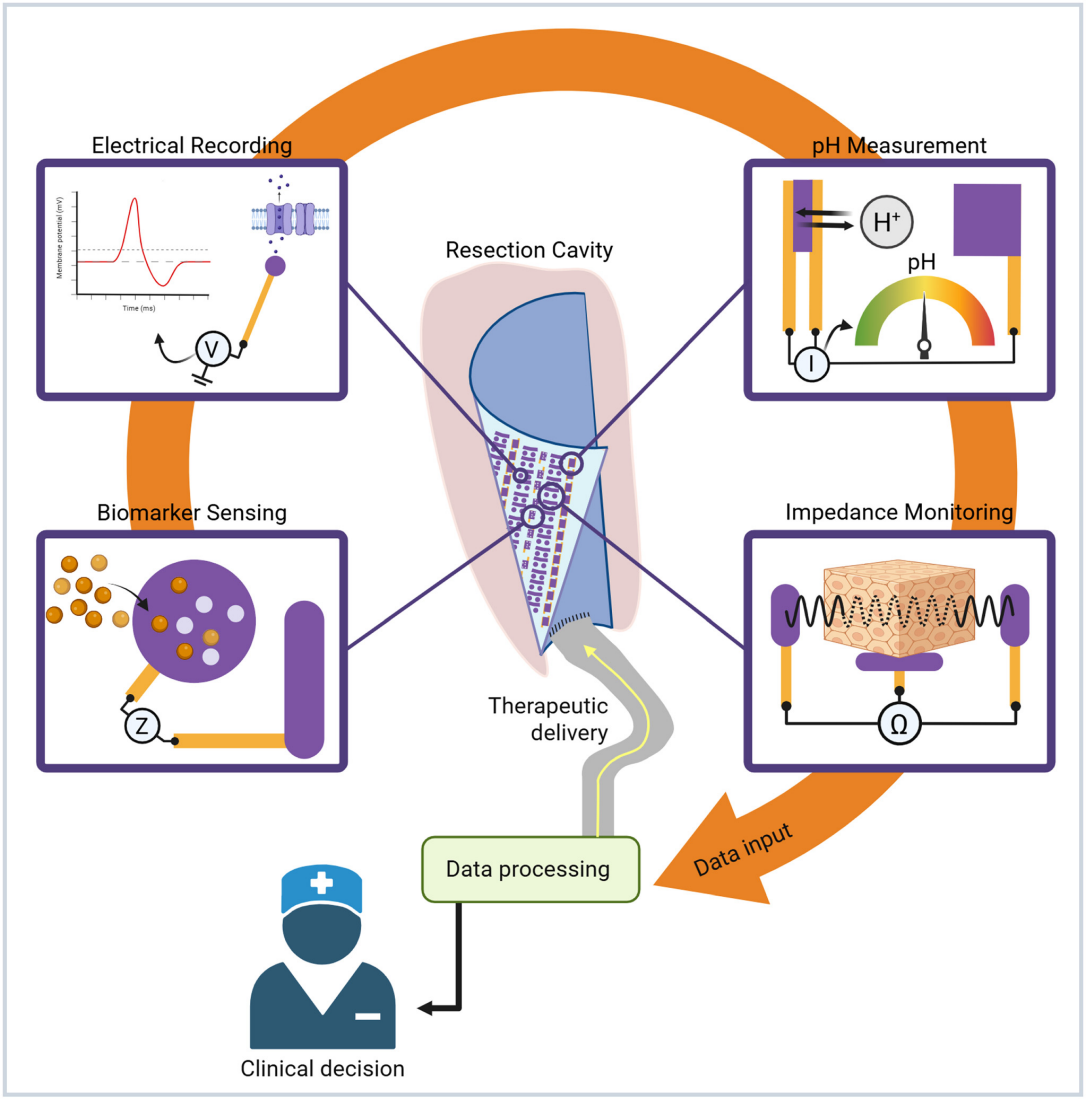
Schematic demonstrating proposed multimodal measurements feeding into precision delivery of therapeutics as well as offering key clinical data output for clinicians to aid in decision making. Created in BioRender, C. Chapman (2026) https://BioRender.com/95ak5zb.

The full realization of a closed-loop system requires integration with an appropriate therapeutic output. Several strategies could be coupled with implantable biosensors to achieve this goal, including novel modalities like electrotherapies, drug delivery platforms, or even immunotherapeutic interventions. Utilizing the physiological signals and data analytics discussed there is a clear way forward toward a combined multisensor approach to infer tumor state and decide on an appropriate treatment level (i.e., electrotherapy duration or drug release amount). In addition to the ability to administer treatments at the first sign of recurrence, the development of a closed-loop system will also arm clinicians with improved information on the current tumor state (Fig. 5). This will facilitate a massive change in post-adjuvant therapy monitoring pathways allowing for MRI scans to be scheduled when needed as well as enable neurooncologists to adjust adjuvant treatments in response to tumor response.

There is a real opportunity to start creating patient centric closed-loop devices for improving the treatment and outcomes for patients with glioblastoma. The success of this opportunity relies on academics, clinicians, and industry startups to joining forces in the field of cancer bioelectronics to create a step-change in the truly personalized treatment for patients with glioblastoma.

## Data Availability

Data sharing is not applicable to this article as no new data were created or analyzed in this study.
